# Brain Control of Plasma Cholesterol Involves Polysialic Acid Molecules in the Hypothalamus

**DOI:** 10.3389/fnins.2017.00245

**Published:** 2017-05-03

**Authors:** Xavier Brenachot, Thomas Gautier, Emmanuelle Nédélec, Valérie Deckert, Amélie Laderrière, Danaé Nuzzaci, Caroline Rigault, Aleth Lemoine, Luc Pénicaud, Laurent Lagrost, Alexandre Benani

**Affiliations:** ^1^AgroSup Dijon, Centre National de la Recherche Scientifique, Institut National de la Recherche Agronomique, Université Bourgogne-Franche ComtéDijon, France; ^2^Institut National de la Santé et de la Recherche Médicale LNC, U1231, Université Bourgogne-Franche Comté, LipSTIC LabEx, Fondation de Coopération Scientifique Bourgogne-Franche ComtéDijon, France

**Keywords:** polysialic acid, hypothalamus, atherosclerosis, HDL, LDL, synaptic plasticity

## Abstract

The polysialic acid (PSA) is a large glycan that is added to cell-surface proteins during their post-translational maturation. In the brain, PSA modulates distances between cells and controls the plasticity of the nervous system. In the hypothalamus, PSA is involved in many aspects of energy balance including food intake, osmoregulation, circadian rhythm, and sleep. In this work, we investigated the role of hypothalamic PSA in the regulation of plasma cholesterol levels and distribution. We report that HFD consumption in mice rapidly increased plasma cholesterol, including VLDL, LDL, and HDL-cholesterol. Although plasma VLDL-cholesterol was normalized within the first week, LDL and HDL were still elevated after 2 weeks upon HFD. Importantly, we found that hypothalamic PSA removal aggravated LDL elevation and reduced HDL levels upon HFD. These results indicate that hypothalamic PSA controls plasma lipoprotein profile by circumventing the rise of LDL-to-HDL cholesterol ratio in plasma during overfeeding. Although mechanisms by which hypothalamic PSA controls plasma cholesterol homeostasis remains to be elucidated, these findings also suggest that low level of hypothalamic PSA might be a risk factor for dyslipidemia and cardiovascular diseases.

## Introduction

Atherosclerosis is characterized by the accumulation of lipoprotein-derived cholesterol in the arterial wall (Hegele, 2009; Goldstein and Brown, 2015). Among lipoproteins, low-density lipoproteins (LDL) play a critical role in the early-onset of the disease. The oxidative modification of LDL promotes the migration of circulating monocytes into the arterial wall and their differentiation into macrophages that in turn scavenge oxidized LDL in an unregulated manner (Tabas et al., 2007). Accordingly, elevated plasma LDL-cholesterol has long been associated with cardiovascular risk and strongly correlates with cardiovascular events (Brown and Goldstein, 1996). On the opposite, high-density lipoproteins (HDL) cholesterol levels are inversely correlated with atherosclerosis but the molecular basis of this relationship is still unclear (Rye et al., 2009). Lowering blood LDL by statins is the first-line strategy against atherosclerosis (Brautbar and Ballantyne, 2011). Additional drugs can be used in combination with statins to further reduce the cardiovascular risk (Libby, 2005). In this way, strategies aiming at increasing HDL-cholesterol are still under development but their net effect on atherosclerosis and cardiovascular events still needs to be demonstrated (Libby et al., 2011). In all cases, current pharmacological treatments target cholesterol metabolism in liver, gut, or in the vascular compartment. A better understanding of how the plasma level of lipoproteins is regulated might provide new opportunities to achieve optimal LDL levels as well as LDL-to-HDL cholesterol ratio.

Many lines of evidence have been accumulated showing a neural control of plasma cholesterol (Perez-Tilve et al., 2011; Bruinstroop et al., 2014; Geerling et al., 2014). Earlier studies performed in rats and rabbits have shown that electrical stimulation (Gunn et al., 1960) or lesion (Bernardis and Schnatz, 1971) in the hypothalamus strongly alter plasma cholesterol. More recently, it has been shown that circulating hormones such as leptin (Vanpatten et al., 2004), insulin (Scherer et al., 2016), and Glp-1 (Parlevliet et al., 2012; Panjwani et al., 2013; Taher et al., 2014), trigger brain-mediated regulation of hepatic cholesterol metabolism. Nutrients such as glucose or oleic acid are also able to regulate VLDL hepatic production via a central action (Lam et al., 2007; Yue et al., 2015). Further pharmacological studies indicate that the melanocortin system, which is sensitive to these hormones and nutrients, is a master regulator of plasma cholesterol. The two main antagonistic components of this system are anorexigenic proopiomelanocortin (POMC) neurons and orexigenic neuropeptide Y/agouti-related protein (NPY/AgRP) neurons. NPY neurons enhance VLDL maturation and secretion (van Den Hoek et al., 2004; Stafford et al., 2008; Bruinstroop et al., 2012; Rojas et al., 2012, 2015) whereas POMC neurons modulate HDL uptake by the liver (Perez-Tilve et al., 2010).

Synaptic inputs on POMC and NPY neurons vary in adult mice, depending on the energy state and changes in fuel availability (Vong et al., 2011; Yang et al., 2011; Benani et al., 2012; Liu et al., 2012). Synaptic plasticity of the melanocortin system likely contributes to the accurate control of energy balance (Benani et al., 2012), and altered synaptic plasticity of POMC and NPY neurons might be a risk factor for metabolic diseases (Pinto et al., 2004; Horvath et al., 2010). We identified the polysialic acid molecule (PSA) as a permissive factor for synaptic reorganization of the melanocortin system during overfeeding (Benani et al., 2012). PSA is a large glycan that is added to specific membrane proteins, including the neural cell-adhesion molecule (NCAM), during post-translational maturation. The attachment of PSA to cell-surface proteins reduces cell interactions and promotes synaptic changes and other plasticity-related events in the brain (Rutishauser, 2008). In the hypothalamus, PSA is involved in many aspects of energy balance including food intake (Benani et al., 2012), osmoregulation (Theodosis et al., 1999), circadian rhythm (Shen et al., 1997; Glass et al., 2000; Fedorkova et al., 2002; Prosser et al., 2003), and sleep (Black et al., 2009). In this work, we investigated whether PSA also contributes to the homeostatic control of plasma cholesterol. To examine this hypothesis, we compared plasma cholesterol-containing lipoproteins levels during overfeeding induced by short-term high-fat diet (HFD) in control mice and PSA-depleted mice. Selective loss of PSA in the hypothalamus was achieved through stereotactic bilateral injection of endoneuraminidase N (endoN), a highly specific bacterial enzyme that cleaves PSA from NCAM residues (Vimr et al., 1984).

## Materials and methods

### Animals

Male C57Bl/6J mice were purchased from the Charles River Laboratories France. Mice were maintained in temperature and humidity controlled rooms on a 12-h/12-h light/dark cycle, with lights on at 07:00 a.m. They were fed either a standard diet (A04; 5.1% lipids; 3.3 kcal/g) or a high fat diet (HFD, reference number: U8954; 22% lipids; 4.4 kcal/g) purchased from Safe, France. Food and water were provided *ad libitum*. For pair-feeding studies, the daily amount of HFD provided to the pair-fed EndoN-treated group (HFDpf mice) was matched to that consumed by the vehicle-treated group fed a HFD (HFD mice). Daily food intake was recorded manually. Experiments were performed on 8-week old mice. All procedures were in agreement with the European Directive 2010/63/UE and were approved by the French Ministry of Research (agreement #00853.01) and by the local ethic committee, i.e., “Comité d'Ethique de l'Expérimentation Animale Grand Campus Dijon” (national identification number: #105).

### Bilateral endoN injections in the hypothalamus

Depletion of PSA in the hypothalamus was achieved by bilateral intra-parenchymal injections of EndoN (0.28 units/side, prepared in phosphate buffer with glycerol, injected volume: 400 nl/side, rate of infusion: 100 nl/min; EuroBio). Injections were performed under isoflurane anesthesia as previously described (Benani et al., 2012; Brenachot et al., 2014). Stereotactic coordinates for injection were: −1.4 mm posterior to the Bregma, ±0.4 mm lateral to the sagittal suture, and −5.6 mm below the skull surface. Control mice received artificial cerebrospinal fluid (aCSF; Tocris Bioscience). Mice were housed individually after surgery. They were kept under controlled temperature and rehydrated with intra-peritoneal injection of saline. They were also injected subcutaneously with buprecare (1 mg/kg) to reduce post-operative pain. Mice were allowed 2 days for recovery before experiment, i.e., before HFD introduction.

### Plasma lipid analyses

Mice were fasted 4 h (with bedding changed and food removed) at 10.00 a.m. and blood samples were collected at 2.00 p.m. under anesthesia with isoflurane. Blood was drawn from the retro-orbital plexus on heparin-containing tubes. Plasma was isolated by a 8,000-rpm spin (7,230 g) at 4°C and samples were stored at −80°C before analysis. Plasma VLDL, LDL, and HDL were isolated by sequential ultracentrifugation as the *d* < 1.006 g/ml, the 1.006 g/ml < *d* < 1.063 g/ml, and the 1.063 g/ml < *d* < 1.21 g/ml fractions, respectively (Hurt-Camejo et al., 2013). Densities were adjusted with KBr solutions. The centrifugation steps for VLDL, LDL, and HDL consisted of 3, 4, and 5-h runs, respectively, at 100,000 rpm (436,000 *g*) in a TLA-100 rotor on a Beckman Optima TLX ultracentrifuge (Palo Alto, CA). Cholesterol levels in total plasma and in lipoprotein fractions were measured enzymatically with a commercially available kit (Cholesterol FS, Diasys, Holzheim, Germany).

### VLDL secretion rate assay

VLDL production rate was determined by measuring the increase in plasma triglyceride levels after injection of an inhibitor of lipolysis as previously described (Sberna et al., 2011). Briefly, 4-h fasted mice were injected intraperitoneally with Poloxamer 407 (P-407, Lasersen, Etampes, France) (1 g/kg body weight). Blood samples were drawn into EDTA-containing tubes and plasma was isolated by centrifugation as described above. Plasma triglycerides levels were determined enzymatically with a commercially available kit (Triglyceride FS, Diasys, Holzheim, Germany). The VLDL triglyceride production rate was calculated from the slope of the curve between 30 and 120 min after P-407 injection and expressed as g/l/min.

### Statistical analysis

All data are expressed as means. Error bars indicate SEM. Multiple comparisons of groups were performed by one-way ANOVA using Prism 5.0 software (GraphPad Software). Newman–Keuls test was used in *post hoc* analyses to compare groups when main effects reached significance. Equality of variances and normality of distribution were checked prior to analysis using Bartlett-test and Kolmogorov–Smirnov-test, respectively. When variances were significantly different or if the data fail the normality test, the Mann–Whitney test was applied. Calculated *p*-values below 0.05 were considered significant. Finally, significant differences between groups were indicated on each graphic representation with a letter and bars without a common letter are significantly different.

## Results

### Plasma lipoproteins profile is rapidly altered upon HFD

**Figure 1:**
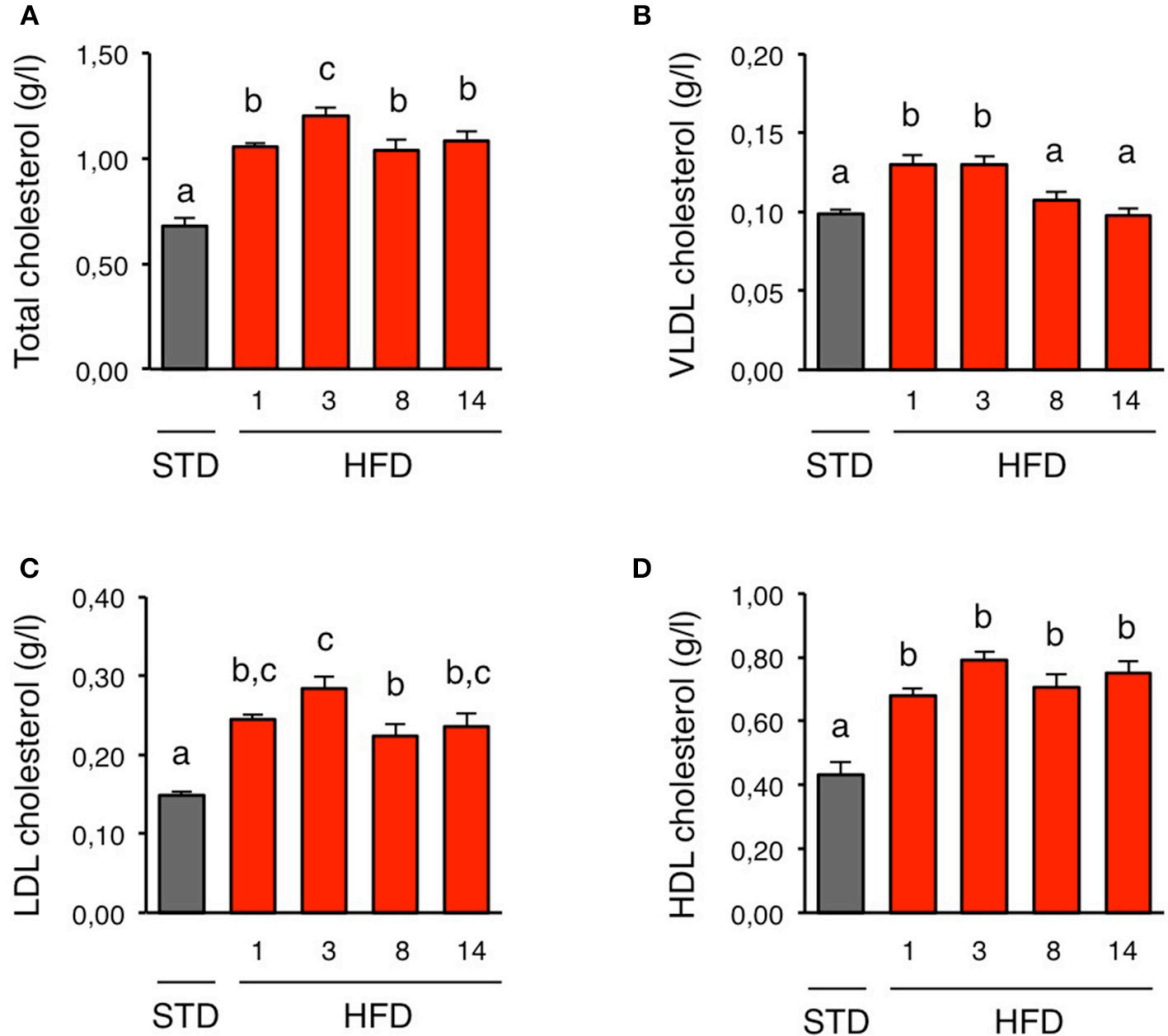
**Plasma lipoproteins profile is rapidly altered upon HFD. (A–D)** Plasma kinetics of total cholesterol, VLDL cholesterol, LDL cholesterol, and HDL cholesterol in mice during a 2 week-high fat diet (HFD; red bars). Control mice were fed with a standard diet (STD; gray bars). *n* = 5–6 for each time-point. Data are presented as mean ± SEM and were analyzed by one-way ANOVA and Newman–Keuls multiple comparison test. Bars without a common letter are significantly different.

We first characterized how plasma cholesterol was regulated upon short-term HFD. For that purpose, we fed mice a HFD for 1 day to 2 weeks and measured total plasma cholesterol and VLDL-, LDL-, and HDL-cholesterol. Control mice were kept on standard diet (STD). HFD consumption for 1 day was sufficient to raise total plasma cholesterol (Figure 1A). This increase was a result of higher VLDL-, LDL-, and HDL-cholesterol levels (Figures 1B–D). Total plasma cholesterol, as well as LDL- and HDL-cholesterol, remained elevated during the 2-week exposure to HFD (Figures 1A,C,D). However, VLDL-cholesterol returned to basal values after 1 week while mice were still kept on HFD (Figure 1B).

### Hypothalamic PSA removal alters plasma lipoprotein homeostasis

**Figure 2:**
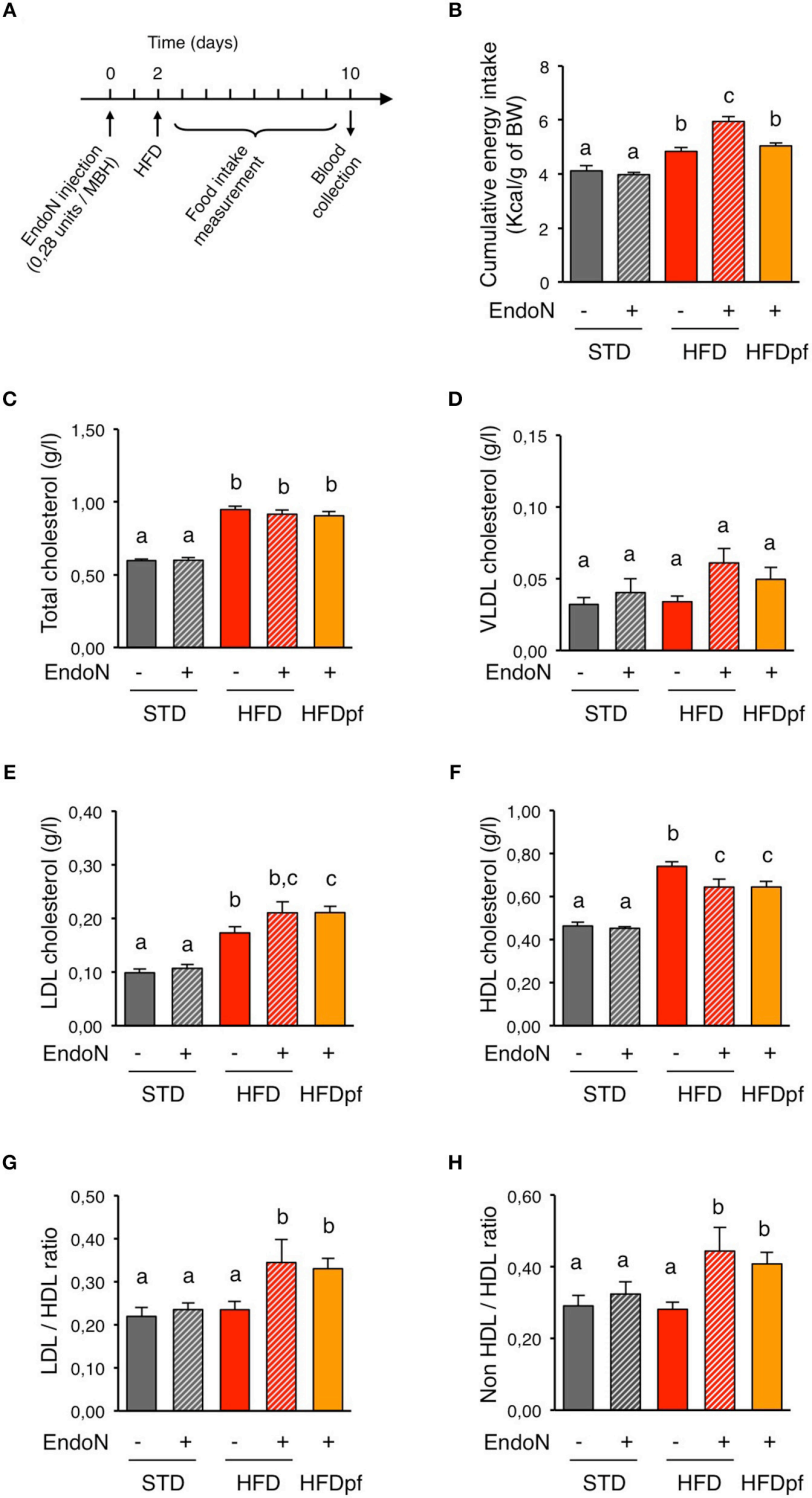
**Hypothalamic PSA removal alters plasma lipoproteins homeostasis. (A)** Picture showing the experimental protocol used to investigate the regulation of plasma cholesterol by hypothalamic PSA. Day 0: To remove hypothalamic PSA, endoN was injected bilaterally in the hypothalamus of mice (0.28 units/side), targeting the mediobasal hypothalamus (MBH). Control mice received artificial cerebrospinal fluid (aCSF). Mice were given 2 days to recover from stereotactic surgery prior to the nutritional challenge. Day 2: Mice were fed either a standard (STD) or a high fat diet (HFD) for 8 days and blood samples were obtained at the end of the experiment (Day 10). **(B)** Effect of intra-hypothalamic endoN injection on cumulative energy intake over a week in mice fed a STD or a HFD. **(C–F)** Effect of intra-hypothalamic endoN injection on plasma total cholesterol, VLDL cholesterol, LDL cholesterol, and HDL cholesterol in mice fed a STD or a HFD for 8 days. **(G,H)** Effect of intra-hypothalamic endoN injection on LDL/HDL and non-HDL/HDL cholesterol ratios in mice fed a STD or a HFD for 8 days. *n* = 10 for STD+aCSF, *n* = 6 for STD+endoN, *n* = 8 for HFD+aCSF, *n* = 8 HFD+endoN, *n* = 7 for HFD pair-fed+endoN. Data are presented as mean ± SEM and were analyzed by nonparametric Mann–Whitney test. Bars without a common letter are significantly different.

To assess the role of hypothalamic PSA in the regulation of circulating cholesterol, we compared levels of plasma cholesterol after 1-week HFD in control and endoN-treated mice (Figure 2A). On STD, daily food intake of control mice that received intrahypothalamic endoN treatment was stable and similar to that of mice receiving aCSF injections. As a result, cumulative energy intake over a week was similar for these two groups (Figure 2B). The absence of behavioral change upon endoN treatment suggests that endoN *per se* does not elicit obvious anorectic inflammatory response. Although this compound did not modify energy intake over a week in mice fed a STD, endoN treatment increased it on HFD (Figure 2B). This typical hyperphagic response induced on HFD by the endoN treatment reveals a PSA-dependent adaptive behavioral response to dietary fat (Benani et al., 2012). To appreciate the contribution of endoN treatment on blood parameters during HFD, irrespective of associated hyperphagia, we pair-fed endoN-treated mice (HFDpf) limiting them to the amount of calories ingested by vehicle-treated control group on HFD (Figure 2B; HFD/aCSF: 4.83 ± 0.14, HFDpf/endoN: 5.04 ± 0.1 kcal/g of body weight over a week). EndoN treatment did not alter plasma cholesterol levels and distribution in STD-fed mice (Figures 2C–F). EndoN treatment did not affect total cholesterol or VLDL-cholesterol in HFD-fed mice too (Figures 2C,D). However, endoN injection in the hypothalamus slightly increased LDL-cholesterol and significantly reduced HDL-cholesterol in HFD-fed mice (Figures 2E,F). Similar effects of endoN treatment on plasma cholesterol were obtained in mice fed a HFD *ad libitum* and in HFD pair-fed mice (Figures 2C–F). Interestingly, LDL/HDL ratio or non-HDL/HDL ratio remained unchanged after 1-week HFD (Figures 2G,H), and endoN treatment did not change these ratios upon STD. However, by affecting both plasma LDL and HDL levels, endoN caused elevation of LDL/HDL and non-HDL/HDL ratios in HFD-fed mice, independently of changes in food intake.

### Hypothalamic PSA does not control hepatic VLDL secretion

**Figure 3:**
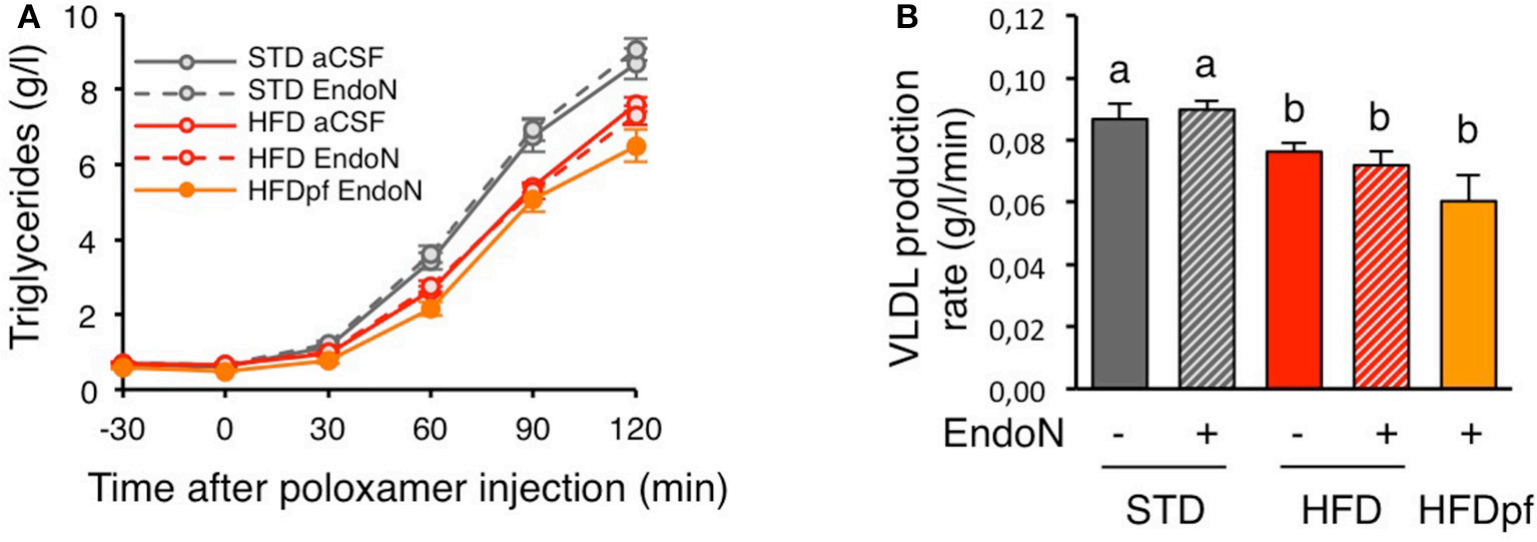
**Hypothalamic PSA does not control hepatic VLDL secretion. (A)** Plasma triglycerides concentration after intraperitoneal injection of poloxamer 407 (*t* = 0) in 4 h fasted mice. Prior to poloxamer injection, mice received bilateral injection of endoN (0.28 units/side) or aCSF in the hypothalamus and were fed a standard (STD) or a high fat diet (HFD) for 8 days, as described in Figure 2A. **(B)** Effect of intra-hypothalamic endoN injection on VLDL production rate in mice fed a STD or a HFD for 8 days. VLDL production rate calculated from the slope of the rise of plasma triglycerides after poloxamer injection. *n* = 11 for STD+aCSF, *n* = 13 for STD+endoN, *n* = 22 for HFD+aCSF, *n* = 16 HFD+endoN, *n* = 6 for HFD pair-fed+endoN. Data are presented as mean ± SEM and were analyzed by one-way ANOVA and parametric Newman–Keuls multiple comparison test. Bars without a common letter are significantly different.

We next sought to determine how hypothalamic PSA controls plasma cholesterol in HFD-fed mice. Because neural circuits in the brain directly control cholesterol metabolism by the liver (Perez-Tilve et al., 2010), we investigated the hepatic function after hypothalamic PSA manipulation. We assessed hepatic production of VLDL in mice that received endoN or vehicle in the hypothalamus. As before, mice were fed either a STD or HFD for 1 week, or were pair-fed on HFD to prevent endoN-induced hyperphagia as a confounding factor. Hepatic VLDL production rate was determined by measuring the kinetics of triglyceride increase after inhibition of VLDL triglyceride hydrolysis by intraperitoneal injection of poloxamer, an inhibitor of lipoprotein lipase (LPL) (Figure 3A). Ingestion of HFD for 1 week decreased VLDL production rate compared to STD-fed mice (Figure 3B). EndoN injection in the hypothalamus did not modify VLDL production rate in STD-fed mice nor during short-term HFD.

## Discussion

In the present study, we report that acute overfeeding induced by HFD consumption rapidly increased plasma cholesterol levels in mice. This was noticeable as early as 1 day after HFD introduction. Characterization of major circulating cholesterol-containing lipoproteins revealed that VLDL, LDL, and HDL cholesterol were all elevated after 1 day on HFD. Interestingly, VLDL-cholesterol returned to basal value within a week although mice were still kept on HFD. Moreover, LDL/HDL as well as non-HDL/HDL cholesterol ratio, remained unchanged after 1-week HFD. These results support the existence of homeostatic mechanisms that limit the rise of VLDL-cholesterol during acute overfeeding and that maintain a balanced plasma lipoproteins profile. Our findings are in line with previous works that evidenced early adaptation of cholesterol metabolism during short-term HFD, and they further show that such mechanism occurs more rapidly than previously anticipated (Srivastava et al., 1991; Hernández Vallejo et al., 2009; Kahle et al., 2013).

We also found that 1-week HFD caused significant reduction of hepatic VLDL production in mice. Opposite effect has been reported in rats after 3-day HFD (Lam et al., 2007; Yue et al., 2012). Apart from possible inter-species differences, this discrepancy might result from successive and opposite modifications in hepatic lipid metabolism upon HFD. Such sequential effect has been already described for the insulin pathway during the time-course of short-term HFD. Actually, progressive β-cell mass expansion in pancreas upon HFD might compensate early hyperglycemia, glucose intolerance, and insulin resistance (Wang et al., 2001; Ahrén and Pacini, 2002; Winzell and Ahrén, 2004; Lee et al., 2011; Stamateris et al., 2013). Since insulin is a strong regulator of cholesterol metabolism (Sparks and Sparks, 1994; Choi and Ginsberg, 2011), sequential changes in insulin secretion and sensitivity might also explain fluctuations in hepatic VLDL release during 1-week HFD. Short-term HFD is also known to induce gene expression reprogramming in the liver affecting several enzymes and transporters involved in the lipoprotein metabolism. For instance, coordinated down-regulation of *Acc, Fas*, and *Hmgcr*, occurs in mouse liver after 1-week HFD, suggesting a reduction of VLDL synthesis on HFD (de Fourmestraux et al., 2004; Hernández Vallejo et al., 2009). These molecular data are consistent with a drop in VLDL release seen in this study. Altogether, these results support the concept that metabolism adapts to compensate for acute cholesterol overload. Such homeostatic response likely results from complementary mechanisms taking place in various organs or tissues. Indeed, 1-week HFD is sufficient to change postprandial release, composition and size of chylomicrons by modulating intestinal lipid metabolism (Hernández Vallejo et al., 2009). Early transcriptional regulation of fatty acid metabolism-related enzymes has been observed in jejunum and duodenum upon 3-day HFD, indicating that the intestine is highly reactive to dietary fat (Clara et al., 2016). In the skeletal muscle, HFD suppresses lipogenic genes within a week and increases oxidative metabolism and markers of type I fiber, showing the metabolic adaptability of this tissue too (de Fourmestraux et al., 2004; Wilson et al., 2007; de Wilde et al., 2008). Similar transcriptional effects also occur in the adipose tissue (Voigt et al., 2013). Circuits in the central nervous system control most, if not all, peripheral metabolic responses that are triggered during energy imbalance (Morton et al., 2006; Myers and Olson, 2012). In this work, we bring the first evidence that hypothalamic PSA, a modulator of neuronal function, is critical to maintain LDL-to-HDL ratio upon HFD consumption on the short term. Given the role of PSA in the control of synaptic plasticity (Rutishauser, 2008), these findings suggest that blood cholesterol homeostasis might be regulated via neuronal rewiring within hypothalamic circuits. Above all, this shows that hypothalamic PSA coordinates many aspects of metabolism and energy balance including peripheral lipid metabolism as well as food intake (Benani et al., 2012), osmoregulation (Theodosis et al., 1999), circadian rhythm (Shen et al., 1997; Glass et al., 2000; Fedorkova et al., 2002; Prosser et al., 2003), and sleep (Black et al., 2009). However, mechanisms by which hypothalamic PSA affects plasma levels of LDL and HDL cholesterol remains to be elucidated. Current models suggest that brain controls circulating cholesterol in a neuroendocrine feedback loop through autonomic effects on liver function (Perez-Tilve et al., 2011; Bruinstroop et al., 2014; Geerling et al., 2014). In particular, it has been shown that the melanocortin system regulates hepatic lipoproteins production and uptake via sympathetic innervation (Bruinstroop et al., 2012; Rojas et al., 2015). Nevertheless, removal of hypothalamic PSA did not alter hepatic VLDL secretion. Thus, to affect plasma levels of LDL and HDL *via* the liver, hypothalamic PSA would act on others hepatic mechanisms that have not been investigated in this work, such as cholesterol uptake, catabolism, or excretion. Since the brain also controls lipid uptake in white adipose tissue and intestinal chylomicron production (Nogueiras et al., 2007; Farr et al., 2015), it is conceivable that hypothalamic PSA modulates lipoprotein metabolism other than in the liver.

Neuronal circuits involved in the PSA-mediated regulation of blood cholesterol metabolism have not been investigated in this study. Previous studies from our group indicate that endoN injections lead to a complete loss of PSA in the whole hypothalamus (Benani et al., 2012). Investigation of circuits underlying the brain control of peripheral lipid metabolism reveals several neuronal targets and signaling pathways located in the hypothalamus, including NPY, POMC, MCH, and glutamatergic neurons (van Den Hoek et al., 2004; Nogueiras et al., 2007; Stafford et al., 2008; Perez-Tilve et al., 2010; Bruinstroop et al., 2012; Rojas et al., 2012; Yue et al., 2012; Imbernon et al., 2013; Rojas et al., 2015). These pharmacological studies show a high degree of specialization among the neuronal circuits that control peripheral lipid metabolism. Actually, distinct neuronal sub-populations are engaged in either adipocyte or hepatocyte metabolism modulation (Imbernon et al., 2013). Furthermore, hypothalamic pathways seem to control VLDL production and HDL uptake separately (Stafford et al., 2008; Perez-Tilve et al., 2010). Since endoN treatment is not cell-selective, we can suppose that PSA-dependent regulation of plasma cholesterol likely integrates inputs from different neuronal circuits. In addition, astrocytes located in the hypothalamus, which also express PSA (Pierre et al., 2001) and control several aspects of energy homeostasis (García-Cáceres et al., 2012; Argente-Arizón et al., 2015), might be involved in PSA-dependent regulation of peripheral lipoproteins metabolism as well.

Lipoproteins profile and metabolism differ in the peripheral circulation and the central nervous system (Pfrieger and Ungerer, 2011; Mahley, 2016). In the adult, only HDL particles are found in the brain. This peculiar feature in lipoproteins reflects the isolation of the brain from the periphery by the blood–brain barrier and the specific metabolism of lipoproteins in the brain. In particular, astrocytes are responsible for the largest production of the most abundant apoE-containing HDL-like lipoproteins that redistribute lipids in the central nervous system. Further studies are needed to examine the potential role of brain PSA, whose expression declines in Alzheimer's disease (Murray et al., 2016), in the maintenance of cholesterol homeostasis in the central compartment as well.

To conclude, this work uncovers a new role for hypothalamic PSA in modulating plasma lipoprotein profile. Although we show that hypothalamic PSA is beneficial by circumventing elevation of LDL-to-HDL cholesterol ratio during overfeeding, neuronal pathways and peripheral mechanisms engaged in this control remain to be elucidated. Nonetheless, our findings suggest that reduced level of hypothalamic PSA might be a risk factor for dyslipidemia and cardiovascular diseases.

## Author contributions

Study conception and design: XB, TG, LL and AB; Acquisition of data: XB, TG, EN, VD, AmL, DN, CR and AlL; Analysis and interpretation of data: XB, TG, LL and AB; Drafting of manuscript: TG, VD, LP, LL and AB.

## Funding

This work was supported by the Centre National de la Recherche Scientifique (CNRS), the Institut National de la Santé et de la Recherche Médicale (INSERM), the Université de Bourgogne-Franche Comté (grant BQR), the Agence Nationale de la Recherche (ANR; ANR-13-JSV1-0003-01), and by a French Government grant managed by the ANR (Program “Investissements d'Avenir” ANR-11-LABX-0021-01-LipSTIC LabEx).

### Conflict of interest statement

The authors declare that the research was conducted in the absence of any commercial or financial relationships that could be construed as a potential conflict of interest.
